# A stereotaxic, population-averaged T1w ovine brain atlas including cerebral morphology and tissue volumes

**DOI:** 10.3389/fnana.2015.00069

**Published:** 2015-06-04

**Authors:** Björn Nitzsche, Stephen Frey, Louis D. Collins, Johannes Seeger, Donald Lobsien, Antje Dreyer, Holger Kirsten, Michael H. Stoffel, Vladimir S. Fonov, Johannes Boltze

**Affiliations:** ^1^Department of Cell Therapy, Fraunhofer Institute for Cell Therapy and ImmunologyLeipzig, Germany; ^2^Faculty of Veterinary Medicine, Institute of Anatomy, Histology and Embryology, University of LeipzigLeipzig, Germany; ^3^McConnell Brain Imaging Centre, Montreal Neurological Institute and Hospital, McGill UniversityMontreal, QC, Canada; ^4^Department of Neuroradiology, University Hospital of LeipzigLeipzig, Germany; ^5^Translational Centre for Regenerative Medicine, University of LeipzigLeipzig, Germany; ^6^Faculty of Medicine, Institute for Medical Informatics, Statistics and Epidemiology, University of LeipzigLeipzig, Germany; ^7^LIFE Center (Leipzig Interdisciplinary Research Cluster of Genetic Factors, Phenotypes and Environment), University of LeipzigLeipzig, Germany; ^8^Division of Veterinary Anatomy, Vetsuisse Faculty, University of BernBern, Switzerland; ^9^Neurovascular Regulation Laboratory at Neuroscience Center, Massachusetts General Hospital and Harvard Medical SchoolCharlestown, MA, USA

**Keywords:** brain, atlas, tissue segmentation, structural MRI, sheep, stereotaxy, SPM

## Abstract

Standard stereotaxic reference systems play a key role in human brain studies. Stereotaxic coordinate systems have also been developed for experimental animals including non-human primates, dogs, and rodents. However, they are lacking for other species being relevant in experimental neuroscience including sheep. Here, we present a spatial, unbiased ovine brain template with tissue probability maps (TPM) that offer a detailed stereotaxic reference frame for anatomical features and localization of brain areas, thereby enabling inter-individual and cross-study comparability. Three-dimensional data sets from healthy adult Merino sheep (*Ovis orientalis aries*, 12 ewes and 26 neutered rams) were acquired on a 1.5 T Philips MRI using a T1w sequence. Data were averaged by linear and non-linear registration algorithms. Moreover, animals were subjected to detailed brain volume analysis including examinations with respect to body weight (BW), age, and sex. The created T1w brain template provides an appropriate population-averaged ovine brain anatomy in a spatial standard coordinate system. Additionally, TPM for gray (GM) and white (WM) matter as well as cerebrospinal fluid (CSF) classification enabled automatic prior-based tissue segmentation using statistical parametric mapping (SPM). Overall, a positive correlation of GM volume and BW explained about 15% of the variance of GM while a positive correlation between WM and age was found. Absolute tissue volume differences were not detected, indeed ewes showed significantly more GM per bodyweight as compared to neutered rams. The created framework including spatial brain template and TPM represent a useful tool for unbiased automatic image preprocessing and morphological characterization in sheep. Therefore, the reported results may serve as a starting point for further experimental and/or translational research aiming at *in vivo* analysis in this species.

## Introduction

Neuroimaging and accurate stereotaxic neuronavigation is increasingly important in clinical and translational neuroscience. A spatially standardized coordinate system for human subjects based on a single post-mortem brain was defined almost three decades ago (Fox et al., 1985; Talairach and Tournoux, 1988). However, the Talairach coordinate system posed limitations with respect to morphological representation of larger study populations and was therefore thoroughly amended by the Montreal Neurological Institute (MNI). The stereotaxic space was redefined (Evans et al., 1994) and data sets were linearly scaled to the original Talairach space (Collins et al., 1994, 1995). Later, an even more detailed standard reference system was created (ICBM 452 template, Mazziotta et al., 2001; Lancaster et al., 2007). Adding to this, non-linear transformation algorithms (Klein et al., 2009) and precise population-averaged high-resolution templates for adults and infants were developed (Fonov et al., 2011), enabling the template to serve as an objectified, morphological, and spatial reference for image analysis of the human brain.

Similar templates have been developed for rodents (Calabrese et al., 2013; Nie et al., 2013), rabbits (Munoz-Moreno et al., 2013), canines (Tapp et al., 2006; Datta et al., 2012), and non-human-primates (Newman et al., 2009; Frey et al., 2011), being of utmost value for preclinical and translational studies. However, atlases are lacking for other species including sheep. This is a severe limitation since sheep are increasingly recognized in several fields of translational neuroscience. Compared to other species such as the widely used lissencephalic rodents the larger brain size and body weight (BW) of this gyrencephalic species predestine sheep for translational imaging procedures using clinical scanners (Forschler et al., 2007; van der Bom et al., 2013; Beuing et al., 2014). Considering cerebral perfusion the angioarchitecture of the ovine cerebral venous system is highly similar to humans in contrast to dogs (Hoffmann et al., 2014). The extracerebral arterial system include a ruminant specific vascular network (*Rete mirabile epidurale rostrale*), which may be serve as malformation model (Qian et al., 1999). Sheep are robust and unassuming compared to dogs and non-human primates, especially during long term studies of cerebral injuries, which may be the result of the lower crossover rate of the *decussation pyramids* tracts. Therefore, sheep play an important role in translational neuroscientific research including pain research (Gierthmuehlen et al., 2014), signal processing (Flouty et al., 2013), pediatric (Finnie et al., 2012) and adult traumatic brain injury (Grimmelt et al., 2011), cerebrovascular physiology (Ashwini et al., 2008; Truong et al., 2014), global (Jellema et al., 2013) and focal cerebral ischemia (Boltze et al., 2008; Wells et al., 2012), as well as research on novel therapeutics (Boltze et al., 2011; Terpolilli et al., 2012).

A quality brain template should (i) provide high spatial resolution, (ii) be probabilistic (i.e., represent the population anatomy rather than one individual), and (iii) feature a standard coordinate system (Van Essen and Dierker, 2007). Individual scans should be characterized in detail, e.g., to allow further observations, ultimately improving generalizability. Segmentation procedures (Zhang et al., 2001; Ashburner and Friston, 2005) play a key role in statistical image analysis, since manual and even semi-automatic procedures are subjective, labor-intensive, impractical for processing large data sets, and are often non-reproducible (Zijdenbos et al., 1998). Automated brain tissue classifications can overcome these limitations but many techniques require the *a priori* probability to be registered to a template which in turn is aligned to standard stereotaxic coordinates. Until now, neither an ovine population-averaged MR template within a stereotaxic space nor corresponding tissue probability maps (TPM) have been reported. Here we present a spatially unbiased, non-linearly transformed, population-averaged ovine magnetic resonance (MR) brain template. Gray (GM) and white matter (WM), as well as cerebrospinal fluid (CSF) probability maps were developed for intensity-based automated segmentation, addressing the need for a simple and robust framework. To this end, the capability of image pre-processing with the widely used statistical parametric mapping (SPM8) software was tested in a cohort, and the ovine cerebrum was characterized quantitatively.

## Materials and methods

### Ethical approval

All experiments were approved by the Federal Animal Welfare board in Leipzig, Germany. Of note, all MR imaging (MRI) data sets were obtained from studies conducted for different purposes (animal license numbers TVV33/09, TVV09/11, TVV33/12, and W04/19). For those studies, sheep were subjected to stereotactic surgery using a frameless stereotactic device (Rogue Research Ltd., Canada, Dreyer et al., 2012). The procedures required structural/anatomical MRI data sets for neuronavigation which were used for the herein presented study. Brain specimens were obtained from the respective animals upon termination of the respective experiments, omitting the necessity to scarify any animal specifically for this study.

### Study population and pre-test assessment

Thirty eight, hornless Merino sheep (Ovis orientalis aries; 26 rams and 12 ewes) were used in this study. Rams were neutered between 6 and 8 months of age to improve handling and to reduce hierarchical conflicts within the flock. All animals were housed in unisex groups with *ad libitum* access to food and water. Animals were allowed to pasture outside once per day. Food, but not water restriction, was applied for 12 h prior to each imaging session.

To identify subjects with potential abnormalities, a detailed hemogram, physiological assessment (heart rate, breathing frequency, rectal temperature, and BW) and a neurological score (behavioral phenotyping, see Supplementary Information) were taken from each animal prior to enrollment. Animals violating physiological norms in any test item were excluded from the experiment.

### Anesthesia and MR imaging

Subjects were anesthetized by intravenous injection of 4.0 mg × kg^−1^ BW ketamine (Medistar Ltd., Holzwickede, Germany), 0.1 mg × kg^−1^ BW xylazine (Ceva Sante Animal Ltd., La Ballastiére, France) and 0.2 mg × kg^−1^ BW midazolame (Braun Melsungen, Melsungen, Germany) before being intubated and transported to the imaging facility. Animals were placed in prone position and anesthesia was maintained by mechanical respiration (900D, Siemens, Germany) with 2% isofluran (WDT eG, Garbsen, Germany) throughout imaging. MRI scans were performed on a 1.5 T clinical scanner (Gyroscan Intera, Philips). A MR T1 weighted sequence was acquired from each subject [3D T1 FFE series (incoherent gradient echo (RF spoiled) sequence), in-plane resolution 0.39 × 0.39 mm, slice thickness 1.0 mm (voxel size 0.39 × 0.39 × 1.0 mm), acquisition matrix: [0 256 229 0], TE: shortest, TR: 25 ms, Flip Angle 30, recon matrix: 512, slices 110, number of averages: 3, Percent Phase Field of View: 79.7, Pixel Bandwidth: 149.665, SENSE: yes, coil: sense flex medium fixed over both hemispheres, acquisition time: 19 min]. The slab of the MRI sequence was oriented perpendicular to the z-axis of the scanner. Thereafter, animals were transported back to their stable for post-imaging wake up and recovery. Manual ventilation was applied during transportation (about 5 min one way).

### T1w volume template creation

For generation of a T1w template, MR image processing was performed using the MINC toolkit version 1.9.05, available from an online open access source (http://www.bic.mni.mcgill.ca/ServicesSoftware/ServicesSoftwareMincToolKit).

The following image pre-processing steps were applied to 15 randomly selected individual data sets, from which one subject was randomly chosen as a reference: (1) initially, an intensity non-uniformity correction using the N3 algorithm (Sled et al., 1998) was applied to exclude remaining intensity inhomogeneity artifacts which could not be prevented by the CLEAR procedure (“Constant LEvel AppeaRance” as implemented in the SENSE approach of the Gyroscan Intera Philips MR); (2) linear intensity normalization to a range between 0 and 100 using histogram matching, followed by (3) a linear rigid body registration (three rotations and three translations) toward the reference scan. The latter was applied after manual identification of anatomical landmarks by a trained expert that includes the center of the left/right eyeball, the most rostral and caudal poles of both hemispheres, the intersection of the cruciate sulcus and the longitudinal fissure, as well as the anterior (AC) and posterior commissure (PC). (4) Image resampling into the common space of the reference scan was applied with a uniform voxel size of 0.25 mm^3^ using 4th order B-spline interpolation. (5) The final visual inspection of the images resulted in the exclusion of one animal because of misleading registration due to a very large frontal sinus.

The resulting images were used for a three-step average template generation process as described elsewhere (Fonov et al., 2009). In brief, the transformation matrices calculated in one step were applied to the appropriate scan in order to resample the image and where then used for the next step. The following processing steps were performed: (6) a linear left-right symmetric rigid body registration using eight iterations. The rigidly transformed average (Mean_rig_) was created. Then, (7) a left-right symmetric fully-affine (12 parameters) iterative registration procedure was applied to each input scan using 8 iterations followed by calculation of the average (Mean_affine_). (8) A left-right symmetric non-linear registration including 4 iterations with 4 mm steps, 4 iterations with 2 mm steps and 4 iterations with 1 mm steps was applied to each scan before the final, non-linear transformed average was generated (Mean_nl_).

The standard deviation for each voxel was calculated from the averages Mean_rig_, Mean_affine_, and Mean_nl_. All anatomical averages and standard deviations were converted into NIFTI-1 format with 32 bit floating-point voxels.

### Quality of the T1W population-averaged template

Quality of the retrieved averages and individual original image volumes was estimated by calculating signal-to-noise (SNR) and contrast-to-noise (CNR) ratios according to equation (1)

(1) $$SNR=\frac{mean_{ROI}^{gV}}{sd}\;CNR=\frac{mean_{WM}^{gV}-mean_{GM}^{gV}}{\sqrt[{2}]{\left(var_{WM}+var_{GM}\right)}}$$

where *gV* is the gray value, *sd* the standard deviation, *var* the variance, and ROI the region of interest. In contrast to descriptions in the literature, the ROI defines the complete tissue classes (GM and WM) based on tissue masks. These were retrieved individually from each subject, while tissue masks from the final template (Mean_nl_) were used to calculate the parameters in Mean_rig_ and Mean_affine_. Therefore, the SNR represents the homogeneity of gray values within a tissue class while CNR depict the mean gray value difference between WM and GM.

### Morphological characteristics and tissue preparation

Fourteen brain specimens were used for morphological investigation. Ten brain samples were perfused and immersed with 4% paraformaldehyde. Subsequently, brains were manually cut into equally spaced, 4 mm thick coronal slices. In four samples, vital staining by 0.5% triphenyl tetrazolium chloride (TTC) with phosphate-buffered-saline (pH 7.4) for 45 min was performed immediately after dissection and slicing to increase the contrast between gray matter (stained) and white matter tracts (unstained). All brains and brain slices were photographed (Nikon DX 100, Japan).

The 3D viewer plugin integrated in Fiji (F is just ImageJ, http://fiji.sc/Fiji, Schindelin et al., 2012) was used for GM and WM surface reconstruction. Additionally, the plugin volume viewer V2.01 (Barthel, Internationale Medieninformatik, HTW Berlin, Germany, http://imagej.net/plugins/volume-viewer.html) was utilized for segmented brain tissue rendering. Labeling of structures identified in the brain samples and the averaged T1w template was performed according to established standards in the neuroanatomical literature (Schmidt et al., 2012) and the Nomina Anatomica Veterinaria (NAV, International Committee on Veterinary Gross Anatomical Nomenclature (ICVGAN), 2012) with the exceptions of the rostral (AC) and caudal commissure (PC). The medial part of the marginal gyrus was assigned to the endomarginal gyrus.

### Cerebral tissue probability maps

TPM were created as reported previously (Evans et al., 1994; Mazziotta et al., 1995). Initially, all rigidly registered scans were resampled to an isotropic voxel size of 0.5 mm^3^. Cerebral GM, WM, and CSF tissue masks from each scan were generated iteratively by a trained expert using a combination of threshold-based segmentation and manual delineation with Fiji in which cerebellum, medulla oblongata and dura mater were removed. Refinement of the tissue masks was performed by using the automated segmentation procedure in SPM8 (Statistical Parametric Mapping, Members & collaborators of the Wellcome Trust Centre for Neuroimaging, UK, Friston et al., 1994) in which the averaged tissue masks served as TPM. Subsequently, the received GM, WM, and CSF tissue masks from each scan were visually inspected and manually improved using Fiji.

Finally, the individual affine and non-linear transformation matrices from the T1w template creation procedure were applied to the appropriate tissue masks. After final non-linear registration, the averages of each tissue mask represent the probability between 0 and 1 that a voxel belongs to the GM, WM, or CSF tissue class. The averages served as TPM and were implemented in SPM8 for further processing.

### Automated segmentation proof of principle in SPM8

The created T1w template and the TPM were used to test the capability of image preprocessing in SPM8 using scans from all 38 animals (primary endpoint). The secondary endpoint of the proof-of-principle study was to quantitatively characterize the ovine cerebrum.

The original, native, untransformed images were reoriented according to the template space and co-registered to the generated T1w template with SPM8 using standard parameters except for a 6th-B-spine interpolation. GM, WM, and CSF masks were retrieved from all animals and sample homogeneity was verified (covariance) using the VBM8 toolbox (Wellcome Department of Cognitive Neurology, Structural Brain Mapping Group, University Jena, Germany, http://dbm.neuro.uni-jena.de/vbm/). All tissue masks were carefully inspected for segmentation errors and remaining non-brain tissue elements.

In addition, the absolute volumes of GM, WM, and CSF (in mL) were determined after segmentation using SPM8. The total brain volume was calculated by the sum of GM and WM. The total brain volume for each subject was divided by the individual's BW in kilograms to calculate relative tissue volumes for GM, WM, CSF, and total brain tissue. Additionally, brain tissue ratios in particular between absolute GM to WM (GM:WM) volume as well as GM (GM:total brain) and CSF volumes to total brain volume (CSF:total brain) were computed.

### Statistics

Group-wise comparisons of the retrieved parameters SNR, CNR, absolute and relative volume, as well as brain tissue ratios (GM:WM; GM:total brain and CSF:total brain) were performed using SysStat version 12 (SysStat Software Inc, San Jose, USA). All data were tested for normality distribution using Kolmogorov-Smirnow and Shapiro-Wilk tests. No relevant deviations from normality were found. When analyzing the data in a multivariate manner, brain tissue volumes were adjusted using a standard linear model with the respective brain volume as dependent variable, and sex, age and bodyweight as independent variables. Thereby, the variance explained by an independent variable was estimated via partial correlation. A *p*-value < 0.05 was considered statistically significant.

## Results

### Study population and pre-test assessment

No animal had to be excluded from the study for normality violation. Subject weight (kg) and age (months) are given in Table 1.

**Table 1:** **Demographic data of subjects used for T1w template creation**.

**Issue/Animal numbers**	**Parameter**	**Mean ± SD**	**25–75% Quartile**	**Min-Max**
T1w template (*n* = 14)	weight (kg)	47.9 ± 5.1	44.0–52.5	40.0–58.0
	age (months)	12.4 ± 3.2	9.5–14.5	8.0–18.0

*SD, standard deviation; min, minimum; max, maximum*.

### Quality of the T1W population-averaged template

The template represents the intensity and spatial positioning of the averaged anatomical brain structures from each subject. None of the samples included mirroring, aliasing or motion artifacts. Further, bias inhomogeneity, magnetic susceptibility and Gibbs/truncation artifacts were minimal.

**Figure 1:**
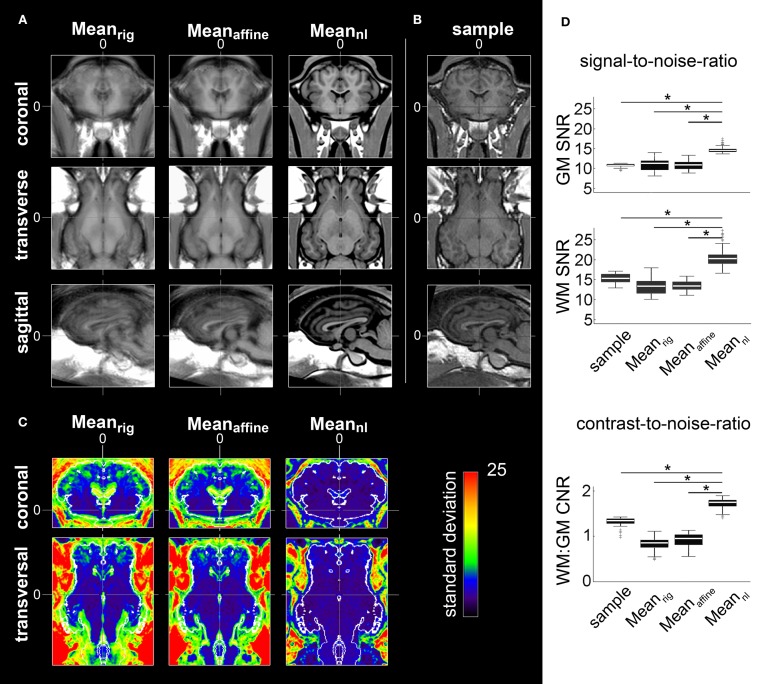
**Averages after rigid body (Mean_rig_), affine coregistering (Mean_affine_), and the final non-linear transformed template (Mean_nl_). (A)** All images have an isotropic voxel size of 0.25 mm and were registered according to ovine stereotactic space for visual comparison of the respective procedure's results. The high degree of aligment within the Mean_nl_ led to an improved boundary delineation and enhanced detail resolution. **(B)** Coronal, transverse, and sagittal planes of an individual brain MRI sample for comparison. **(C)** Coronal and transverse planes showing standard deviation of Mean_rig_, Mean_affine_, and Mean_nl_. The standard deviation inside the cranial cavity decreased after affine and non-linear registration. A restricted RGB lookup table was applied for better visualization of the values for each voxel inside the brain cavity. Additionally, the contour (white) of the segmented brain tissue of the template was overlaid. Stereotaxic coordinates are given in mm. **(D)** Comparison of SNR and CNR: compared to Mean_rig_, Mean_affine_, and the sample GM and WM SNRs of Mean_nl_ were significantly increased. Furthermore, the CNR significantly increased in Mean_nl_ after Mean_rig_ and Mean_affine_ transformation. The comparison between the sample data and Mean_nl_ revealed a significantly increased CNR in the non-linear transformed population-averaged brain template (^*^p < 0.05. “+” depict outliers). The box plots show 95% confidence interval, 25/75% quartile and median. GM, gray matter; WM, white matter; CSF, cerebrospinal fluid; SNR, signal-to-noise-ratio; CNR, contrast-to-noise-ratio.

The final, non-linearly transformed ovine brain template had a much better resolution than individual samples (Figures 1A,B). In contrast, both the rigidly and affine transformed averages were blurred and featured insufficient detail resolution. Compared to the individual T1w samples, the nonlinearly averaged template Mean_nl_ had a significantly superior GM and WM SNR (p < 0.05; Figure 1B). The standard deviation of each voxel inside the cranial cavity decreased after affine and non-linear registration (Figure 1C). As expected, GM and WM SNR in Mean_rig_ and Mean_affine_ were significantly lower than in the non-linearly averaged template (*p* < 0.05, Figure 1D).

Furthermore, the CNR increased significantly during the registration process which contributed to the improved detail resolution of the final non-linear transformed template (*p* < 0.05). The CNR obtained from linearly transformed Mean_rig_ and Mean_affine_ showed less contrast compared to CNR of individual samples (*p* < 0.05, Figure 1D).

### The ovine coordinate system

**Figure 2:**
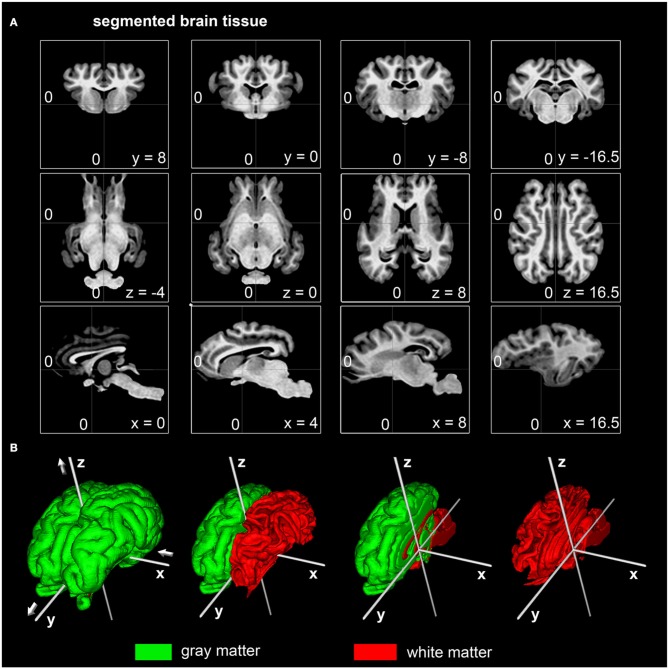
**Stereotaxic coordinates of the segmented ovine population-averaged template. (A)** The dorsal border of the anterior commissure was the origin of the Cartesian coordinate system (0;0;0) with the posterior commissure in line (y-axis) at coordinates (0;-16.5;0). All coordinates are given in millimeters. **(B)** Illustrations of the surface reconstruction for gray and white matter with the applied stereotaxic coordinate system (white): Values of the x-axis increase from left (negative) to right (positive) while the y-axis increase from caudal (negative) to rostral (positive). Values of the z-axis rise from ventral (negative) to dorsal (positive).

A stereotaxic coordinate system was generated according to MNI template specifications. This resulted in a mid-plane line passing the superior part of the anterior (AC) and the posterior part of the posterior commissure (PC, Figure 2A). The origin of the Cartesian system with the xyz-values (0;0;0) was a vertical line perpendicularly intersecting the superior aspect of the AC. Values of the x-axis increase from left to right while values of the y-axis increase from rostral to caudal. Z-coordinates increase in dorsal direction. All coordinates are given in millimeters (Figure 2B).

### Morphological characteristics

**Figure 3:**
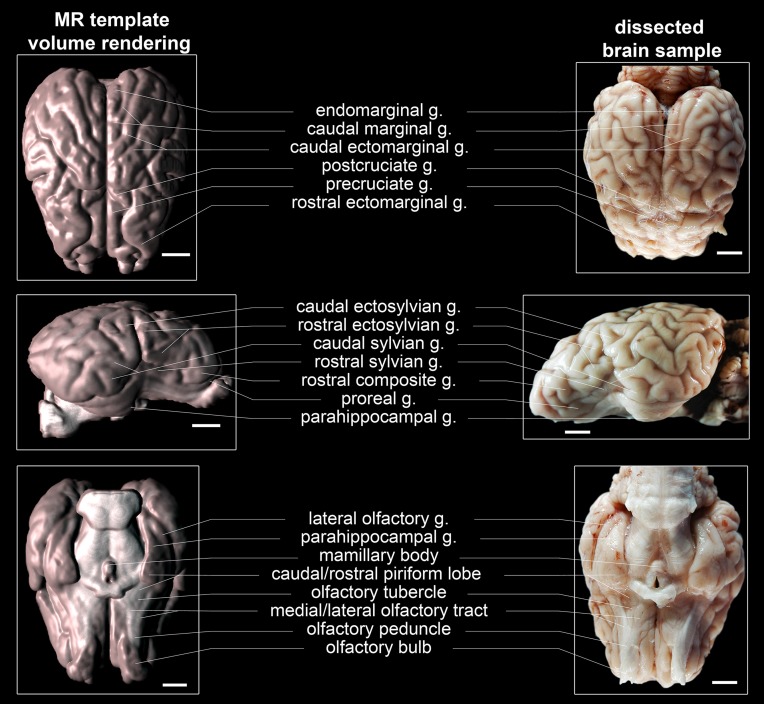
**Topography of volume rendered, population-averaged ovine template, and a fixed specimen**. The most relevant gyri and tractus were labeled in accordance with current neuroanatomical literature (Schmidt et al., 2012) and the Nomina Anatomica Veterinaria [NAV; International Committee on Veterinary Gross Anatomical Nomenclature (ICVGAN), 2012] except the endomarginal gyrus, which is not differentiated from the marginal gyrus according to the NAV. Scale bars: 10 mm.

**Figure 4:**
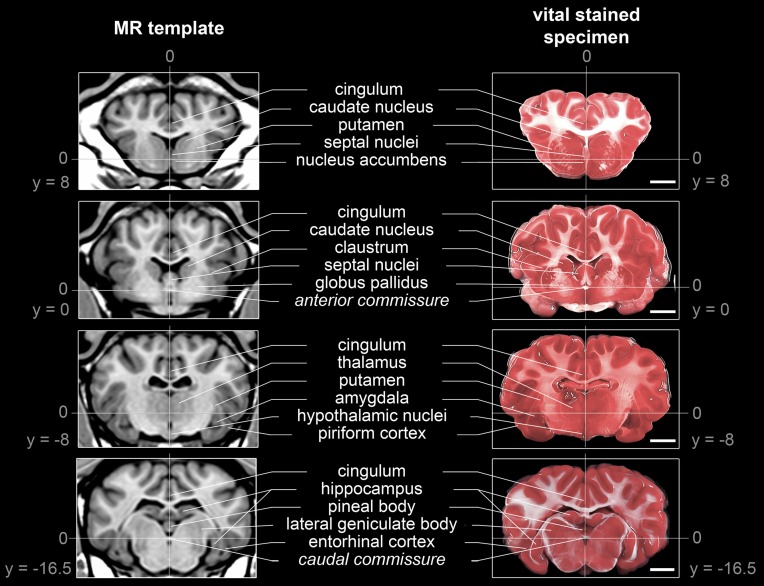
**Comparison of subcortical structure detectability in the population-averaged T1w template and a TTC-stained brain slice**. Structures were selected according to neuro-anatomical reference paper (Cooley and Vanderwolf, 2004; Schmidt et al., 2012): The anterior commissure (AC) as origin of the stereotaxic space [0;0;0] and the posterior commissure (PC) could be identified in both modalities. Note that the coronal planes of the stained slices were not perfectly parallel to the z-axis due to minimal deviations during the slicing procedure. All coordinates are given in mm. Scale bars: 10 mm.

The generated ovine population-averaged template comprised T1w images of 14 subjects. Applying an isotropic voxel size of 0.25 mm allowed the preservation of morphological details. Three-dimensional volume reconstruction of the segmented ovine cerebrum revealed a detailed morphological topography. In both the dissected brain and the rendered volume, primary, secondary and tertiary gyri formations were clearly differentiable (Figure 3). Most relevant subcortical nuclei were equally detectable in TTC-stained coronal brain sections and the T1w template (Figure 4).

### Cerebral TPM

**Figure 5:**
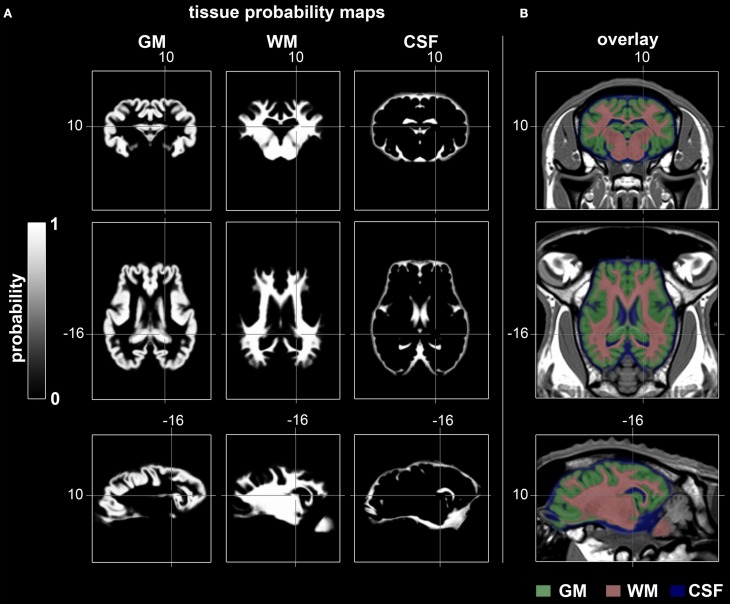
**Coronal, transverse, and sagittal slices visualizing GM, WM, and CSF tissue probabilities. (A)** TPM were created by averaging binary coded, segmented images from the 14 non-linearly registered subjects. Values range from 0 (black) to 1 (white). **(B)** Merged overlay of GM (green), WM (red), and CSF (blue) TPM with the template. Stereotaxic coordinates are given in mm. GM, gray matter; WM, white matter; CSF, cerebrospinal fluid.

TPM for the ovine stereotaxic space were generated from 14 subjects (Figure 5). Tissue probabilities for GM, WM, and CSF tissue classes included cerebral peduncles and pons, but excluded cerebellum and medulla oblongata. The resolution of the TPM with an isotropic voxel size of 0.5 mm allowed a detailed automated segmentation procedure with respect to the target volume.

### Proof of principle for automated segmentation in SPM8

The segmentation procedure of the T1w template resulted in sharply demarcated GM, WM, and CSF masks that matched well with the corresponding tissue in the T1w template (resampled to 0.5 mm isotropic voxel size). Small, false-positive brain tissue adnexae (cruciate region and tentorium sellae) remained in the GM mask, potentially due to misalignment in these CSF-space-limited regions. The resulting volume characteristics (after manual removal of these adnexae) are given in Table 2.

**Table 2:** **Tissue volume characteristics of the ovine T1w template**.

**VOLUME OF THE TISSUE CLASSES (mL)**	
GM	50.3
WM	42.8
CSF	33.9
Total brain volume	93.2
**VOLUME INDICES/RATIO (mL per mL)**	
GM:WM ratio	1.3
CSF:total brain ratio	0.4
GM:total brain ratio	0.6
**VOLUME PER kg BODYWEIGHT (mL per kg)**	
Total brain volume per kg bodyweight	1.9
GM per kg bodyweigth	1.0

*sd, standard deviation; min, minimum; max, maximum; GM, gray matter; WM, white matter; CSF, cerebrospinal fluid; relative tissue volumes were calculated based on the averaged body weight of the used subjects (see Table 1)*.

**Figure 6:**
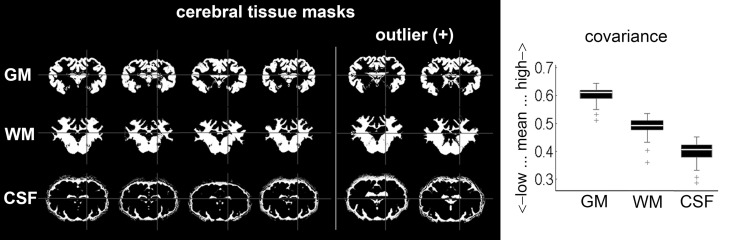
**Mean covariance from automatically segmented tissue masks** **(*n* = 38): six exemplary results (including the two only detected GM; WM and CSF outliers) after segmentation procedure with SPM**. Outliers were subjected to careful visual inspection in order to identify potential non-cerebral tissue adnexae, but no mask was excluded according to pre-set criteria (no significant misclassifications). GM, gray matter; WM, white matter; CSF, cerebrospinal fluid.

Covariance analyses identified two outliers in each tissue class, all in the same animals (Figure 6). However, manual inspection for non-cerebral tissue and artificial segmentation results, including the two outliers, did not warrant exclusion.

The absolute volumes of GM (51.5 ± 4.4 mL), WM (35.6 ± 3.7 mL), and CSF (29.7 ± 3.5 mL) were calculated from the tissue masks. Tissue volumes (per kg BW) were 1.0 ± 0.2 mL (GM), 0.7 ± 0.1 mL (WM), 0.6 ± 0.1 mL (CSF), and 1.7 ± 0.3 mL (total brain). GM:WM ratio was 1.5 ± 0.1, GM to total brain volume was 0.6 ± 0.02, and the relation between CSF and total brain volume was 0.3 ± 0.1. When adjusting on BW and age, the tissue volumes were 51.5 ± 3.9 mL (GM), 35.6 ± 3.2 mL (WM), 29.7 ± 3.3 mL (CSF), and 87.1 ± 6.0 mL (total brain volume).

### Tissue volume analyses considering body weight, age and sex differences

**Figure 7:**
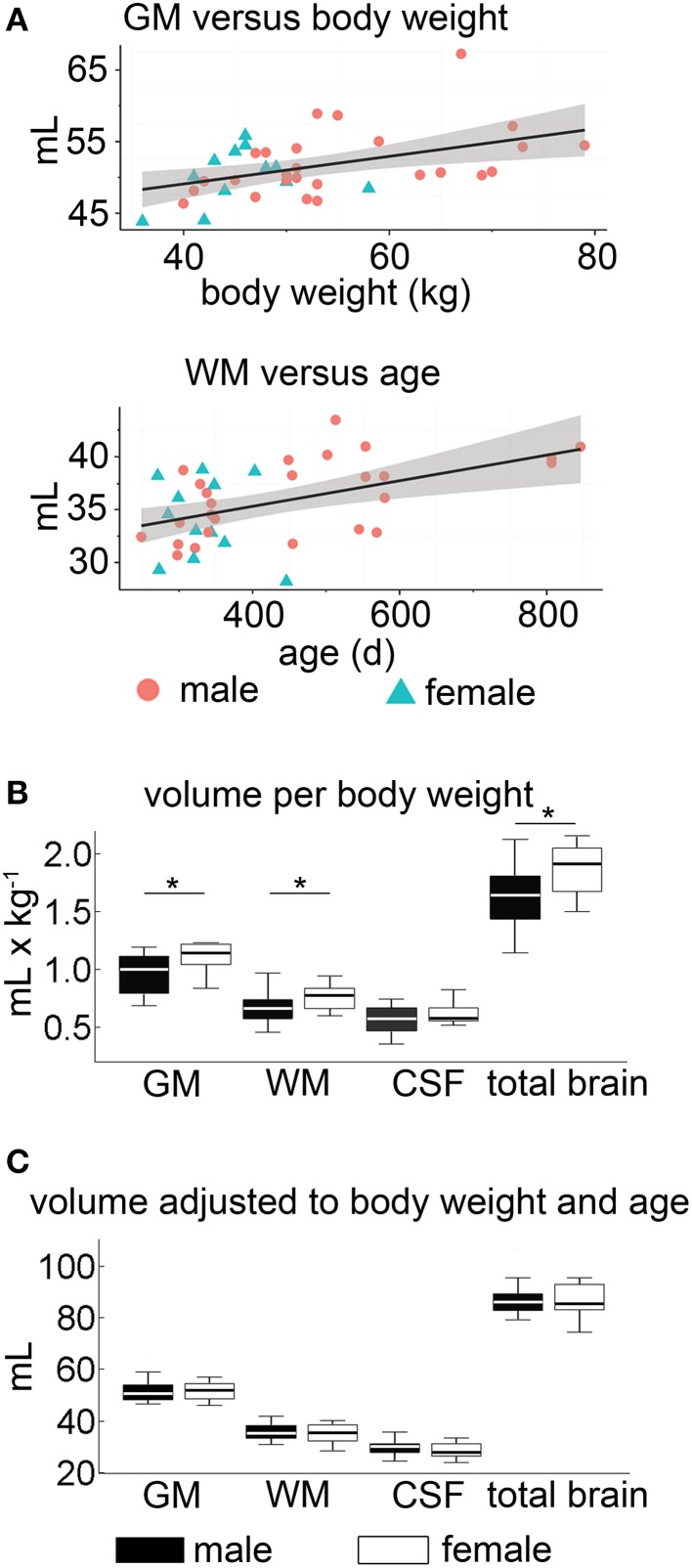
**Volumetric analyses within the proof-of-principle study. (A)** The correlation of body weight on GM and age on white matter WM volumes, respectively, showed a slight positive correlation. **(B)** Sex differences: significant tissue volume differences of GM and WM were observable between male (black) and female (white) sheep when normalized to BW (^*^p < 0.05). **(C)** When simultaneously accounting for the effect of body weight and age in a linear model, no statistical differences of the absolute adjusted brain tissue volume between the sexes were detectable (*p* > 0.05). The box plots show 95% confidence interval, 25/75% quartile and median. GM, gray matter; WM, white matter; CSF, cerebrospinal fluid; BW, body weight.

Demographic data of the study population are given in Table 3. Tissue volumes were analyzed in the entire study population for a relationship between BW, age, and sexual dimorphisms (Figure 7).

**Table 3:** **Demographic data within the proof-of-principle study (*n* = 38)**.

**Parameter**	**Sex**	**Mean ± SD**	**25–75% Quartile**	**Min-Max**
Weight (kg)	All (male/female)	52.5 ± 10.5 (55.6 ± 10.8/45.7 ± 5.5)	45.0–58.0 (48.0–65.0/42.5–48.5)	36.0–79.0 (40.0–79.0/36.0–58.0)
Age (months)	All (male/female)	12.9 ± 4.5 (14.1 ± 4.8/10.3 ± 1.8)	10.0–15.0 (10.0–18.0/9.0–11.0)	10.0–15.0 (8.0–26.0/8.0–14.0)

*SD, standard deviation; min, minimum; max, maximum*.

Overall, the absolute CSF volume was nominally lower in female animals (*p* < 0.05). Comparing the relative tissue volume according to bodyweight (Figure 7B), ewes presented a significantly higher GM volume (*p* < 0.01). A similar difference was observed for relative WM and total brain volumes (*p* < 0.05 each), but not for the relative CSF volume (*p* > 0.05). Note that in our sample the GM:WM ratio did not differ between sexes (*p* > 0.4), as did the GM:total brain (*p* > 0.4) and CSF:total brain ratio (*p* > 0.05). However, no inter-sex differences were found when ratios of brain tissue volumes, adjusted for individual age and bodyweight differences, were used (Figure 7C, Table 4).

**Table 4:** **Brain tissue volume in mL (mean ± standard deviation) stratified for male and female sheep within the proof-of-principle study (*n* = 38)**.

	**Male**	**Female**
GM GM^a^ (mL)	52.1 ± 4.7 51.5 ± 4.2	50.2 ± 3.8 51.5 ± 3.6
WM WM^a^ (mL)	36.3 ± 3.6 35.7 ± 3.0	34.1 ± 3.7 35.3 ± 3.8
CSF CSF^a^ (mL)	30.6 ± 3.4 30.2 ± 3.4	27.8 ± 3.0 28.5 ± 2.9
Total brain volume Total brain volume^a^ (mL)	88.3 ± 6.9 87.2 ± 5.8	84.3 ± 6.7 86.8 ± 6.6
GM:WM	1.45 ± 0.15	1.48 ± 0.13
GM:total brain volume	0.59 ± 0.03	0.60 ± 0.02
CSF:total brain volume	0.35 ± 0.05	0.33 ± 0.04

a *Volume adjusted for the effects of bodyweight and age; GM, gray matter; WM, white matter; CSF, cerebrospinal fluid*.

When absolute tissue volumes of GM, WM, CSF, and total brain volumes were analyzed in a linear model accounting for the linear effect of age, weight, and sexes, no significant effect of sex (*p* > 0.05) was found.

However, in this model, we identified an independent, significantly positive correlation between GM volume and BW, which explained about 15% of the variance of GM. Additionally, we identified an independent, significantly positive correlation between WM and age. The latter explained about 14% of the variance of age (Table 5).

**Table 5:** **Tissue volumes analysis for a relation with body weight, age, and sexual dimorphisms (effect size/*p*-value/partial explained variance)**.

**Parameter**	**Independent variable**		
	**Age**	**Body weight**	**Sex**
GM	−0.001/0.810/-	0.20/0.018^*^/15.3%	0.05/0.980/-
WM	0.01/0.027*/13.6%	0.03/0.630/-	−0.53/0.690/-
CSF	0.00/0.670/-	0.04/0.570/-	−2.16/0.110/-
Total brain volume	0.009/0.290/-	0.24/0.067/-	−0.48/0.850/-

*GM, gray matter; WM, white matter; CSF, cerebrospinal fluid; effect size of independent variables on brain volumes (in mL) is given for an increase of 1 kg, 1 day, and female vs. male for variables weight, age, and sex, respectively*.

* *p < 0.05*.

## Discussion

The major aim of the present approach was to provide a spatial, unbiased standard ovine T1w brain template including TPM for GM, WM, and CSF classifications, and a coordinate system according to Talairach space conventions (implemented in MNI specification). To this end, 14 individual MRI brain samples were aligned to create an unbiased population-averaged brain template representing relevant topographical and morphological structures. Additionally, sex differences and the influence of BW and age on brain and tissue volumes were analyzed.

### The ovine brain template

The reported template provides a number of advantages to investigators interested in ovine brain models. First, it is useful to allocate coordinates of distinct anatomical structures to experimental findings, for example for electrophysiological (Gierthmuehlen et al., 2014) or functional MRI data. Second, it offers a more objective and highly standardized reference system for stereotaxic targeting of anatomical structures (Dreyer et al., 2012; Staudacher et al., 2014) due to the normalization of the created template. Third, the template provides the ground for subsequent creation of a highly precise ovine brain atlas based on a higher sample population.

Spatial location of relevant cortical and subcortical areas using linear and non-linear registration approaches enables matching of individual histological sections to the template (Chakravarty et al., 2006; Yelnik et al., 2007). Moreover, GM, WM, and CSF TPM co-registered with the template can serve as an excellent starting point for highly precise, three-dimensional wrapping procedures (Ashburner, 2007). Such TPMs are also useful for automated segmentation procedures (Ashburner and Friston, 2005; Eskildsen et al., 2012) as voxel-wise-analyses become increasingly important (Ashburner and Friston, 2000). Finally, the reconstructed surface model might be useful for alternative registration procedures and topographical analyses such as cortical surface-based analysis (Dale et al., 1999; Fischl et al., 1999).

### Quality and morphological characteristics

The resolution of most clinical scanners which can accommodate large animals is lower than that provided by high-field small animal scanners. Due to the smaller brain volume in animals compared to humans, the resolution in images obtained by clinical scanners is sometimes poor. Hence, linear inter-subject registration will offer only mediocre improvements. On the other hand, anatomical tissue processing for histology provides far better resolution but is prone to additional problems such as shrinkage or slicing artifacts that may occur during post-mortem tissue preparation. To overcome this dilemma, we relied on additional non-linear transformation algorithms and proper voxel sizes to ensured high detail resolution and contrast. Furthermore, the registration algorithm (Fonov et al., 2009) preserving the bilateral symmetry of the brain, enable future unbiased lateralization studies of the sheep. The resolution of the data could not increased simply by oversampling to 0.25 mm. However, we aimed to minimize sampling errors on individual volumes and averaging multiple oversampled volumes which brings the template reconstruction closer to the Nyquist limit of the data. Finally, the brain template was carefully compared to anatomical preparations, resulting in a relatively high number of details preserved by the non-linear transformation algorithms.

Moreover, we aimed to provide a hitherto unavailable stereotactic reference system for the ovine brain and to identify well-known anatomical structures (Cooley and Vanderwolf, 2004; Schmidt et al., 2012). By referring to individual brain areas with the averaged brain template coordinate system, this may provide the basis for the creation of an even more detailed spatial sheep brain atlas.

### Cerebral tissue probability maps

Many intensity-based segmentation procedures (Ashburner and Friston, 2005) necessitate the *a priori* probability in a defined space to estimate spatial distribution of tissue classification. Therefore, we aligned all subjects to the reference space. The number of subjects required to precisely define the *a priori* probability has not been well investigated so far. With respect to sample size (*n* = 14), our approach may have some limitations compared to equivalent work in humans (152 subjects, Evans et al., 1994), macaques (52 subjects, McLaren et al., 2009), and inbred Wistar rats (30 subjects, Valdes-Hernandez et al., 2011). Importantly, with increased knowledge on anatomical and volumetric variance within this species, additional cases can be added later, continuously increasing the accuracy of the atlas. The approach presented herein can therefore be considered as a starting point to collect this information, which features a sample size that is already large enough to provide a population-averaged sheep brain template. This may represent an advantage to other examples such as the template creation procedure in humans (Talairach and Tournoux, 1988, see introduction section of the manuscript), for which much lower samples sizes (i.e., *n* = 1) were used initially.

Some preprocessing tools such as SPM, BEaST, INSECT (McConnel Brain Imaging Center, Canada, Eskildsen et al., 2012), FSL (FMRIB's Software Library, Analysis Group, FMRIB, UK), and Brainsuite (Laboratory of NeuroImaging and Biomedical Imaging Group, US) exist for brain analyses, each of which has been created for different purposes. Preprocessing gyrencephalic, large animal brains is rarely described in the literature with the exception of non-human primates. Since brain tissue volume differs between human and animals due to volume and shape of the cerebral truncus, the established algorithms may need to be adapted. Beside the herein described methods using SPM, Tapp et al. (2006) described an initial skull stripping approach using the Brain Extraction Tool (BET as implemented in FSL) followed by tissue classification in SPM. FSL was also used to establish a piglet brain atlas including TPM (Conrad et al., 2014) since the software does not require *a priori* probabilities after skull stripping. Furthermore, a SPM toolbox has been developed to preprocess rodent MRI data (http://www.spmmouse.org/). In summary, SPM may provide higher accuracy when compared to FSL and BrainSuite (Kazemi and Noorizadeh, 2014).

Despite of this potential limitation, segmentation procedures with the generated TPM using SPM8 lead to sharply demarcated, homogenous cerebral tissue masks which facilitated further analysis. Since the TPM included male and female subjects with different body weights and ages (see Table 1), an enhanced generalizability of tissue classification may be assumed. However, this may be slightly affected by the marginally imbalanced sex distribution within the template population. Since a significant difference in skull formation between lambs (<6 months) and adult sheep was reported (Rajtova, 1978), the use of the TPM may also be limited to adult sheep. We chose to focus on the widely used and available Merino sheep although potential differences in brain size between sheep breeds have never been investigated. The methods presented here would enable such comparisons.

The data preprocessing approach using SPM8 demonstrated the applicability of the template and the TPM for further image analysis as brain masks from healthy animals were generated. Volumetric characterization of the adult sheep brain is rarely described in the literature which prompted us to investigate global volume ratios which may fuel further investigations of aging, gender differences or atrophy (Nowinski et al., 2011). Furthermore, the relation between CSF and total brain tissue (Palm et al., 2006) was analyzed within the population of healthy subjects and may serve as a starting point for experiments aiming at hydrocephalus formation or global brain atrophy in sheep.

The total brain volume of gyrencephalic mammals such as dogs (77 mL; mesencephalic breeds; Schmidt et al., 2014), non-human-primates (29x2/80/98 mL; Macaque fascicularis/mulatta/nemestrina; McLaren et al., 2009), and sheep (see Table 4) is nearly about 10 fold smaller than human brain volume (1,087/962 mL; male/female; Allen et al., 2003). This should be considered in translational therapeutic studies (STAIR-group, 1999). While a more detailed comparison of brain compartment rations is currently lacking in dog, GM:total brain (see Table 4) in adult sheep is almost comparable with those reported in humans (0.55; Luders et al., 2002) or in non-human-primates (0.58; Chen et al., 2013).

### Tissue volume analyses considering body weight, age and sex differences

Fully automated tissue classifications will not only open the possibility for further structural and functional analyses, but may also shed new light on the relation between age, weight, and sexual dimorphism with cerebral volume measures being of relevance for inter-species comparisons. In particular, when controlling for potential effects of each variable on the other, we identified an independent positive correlation between BW and GM, as well as between age and WM. We cannot exclude an effect of sex on brain volume differing from that described in humans (Allen et al., 2002; Luders et al., 2002). However, the absolute volume of brain tissue does not differ between neutered males and ewes, whereas the body mass-normalized tissue volume is significantly increased in females. Related to this is a recent study indicating the importance of gonadotropine-releasing hormone on cerebral tissue volume of male and female sheep during the first 12 months of life (Nuruddin et al., 2013). The authors described absolute and regional volume differences during puberty between male and female ovine twins which corresponded to those observed in humans (Peper et al., 2011), but did not determine any effect of bodyweight and age. Nevertheless, discussions of sexual dimorphisms from our data require caution due to the fact that neutered rams (neutered at 8 months of age) were used. On the other hand, the effects of sexual hormones on the ovine brain are not well understood, nor are the kinetic or potential “sensitive” time windows. Therefore, our population including rams neutered at a relatively old age may offer a useful starting point to further elucidate the impact of sex hormones on cerebral development in sheep.

### Public data availability

All provided data (template, sample data and TPM) are stored in MINC and NIFTI-1 format. The data can be viewed in most common imaging software including SPM, FSL (Analysis Group, FMRIB, Oxford, UK, Smith et al., 2004) and other packages. The spatial T1w brain template (0.25 and 0.5 mm isotropic voxel size), a 1.5 T MR T1w sample image, and the cerebral TPM are available at (http://www.bic.mni.mcgill.ca/ServicesAtlases/HomePage).

### Conflict of interest statement

The authors declare that the research was conducted in the absence of any commercial or financial relationships that could be construed as a potential conflict of interest.
