# Supplementary material for: A stereotaxic, population-averaged T1w ovine brain atlas including cerebral morphology and tissue volumes
Source: Front Neuroanat. 2015 Jun 4;9:69. doi: 10.3389/fnana.2015.00069 (PMC4455244; doi:10.3389/fnana.2015.00069)
Supplement: Supplementary file 1 [file Table1.DOCX]

**Supplementary information**

**Behavioral phenotyping by Boltze et al. 2008**

The initially performed neurological score is based on the common test system for neurologic dysfunctions in large animals (Oliver et al, 1997) and include

1. state of activity
2. remaining food debris in the mouth
3. torticollis
4. carpus and/or fetlock in partial flexion
5. ataxia/dysmetria
6. Circlic movements
7. hemistanding reaction
8. hopping reaction
9. wheelbarrowing

The score ranges from 0 (no alteration) to a maximum of 27 (stupor/coma).

Oliver, J. E., Lorenz, M. D., and Kornegay, J. N. Handbook of Veterinary Neurology. 3, 1-453. 1997. Philadelphia, WB Saunders Company.

**Supplementary tables**

**Supplementary Table 1:** Subject hemogram, clinical chemistry and clinical parameters of the study population

| **parameter** | **T1w template group**  **(mean±sd)** | **Proof-of principle group**  **(mean±sd)** | **normal values** |
| --- | --- | --- | --- |
| **hemogram** |  |  |  |
| leukocytes (G/L) | 5.2±1.7 | 5.3±1.8 | 5.0–11.0 |
| erythrocytes (T/L) | 10.5±1.5 | 10.5±1.3 | 7.0–11.0 |
| hemoglobine (mmol/L) | 6.9±0.7 | 7.1±0.7 | 5.6–9.3 |
| hematocrit (L/L) | 0.33±0.06 | 0.32±0.04 | 0.27–0.40 |
| MCV (fl) | 30±4.5 | 30±4.1 | 28-40 |
| MCH (fmol) | 0.7±0.1 | 0.7±0.1 | 0.6–0.7 |
| MCHC (mmol/L) | 22±2.2 | 22±1.7 | 19-23 |
| thrombocytes (G/L) | 420±178 | 454±191 | 280–650 |
| neutrophile (G/L) | 2.7±2.6 | 2.9±3.8 | 0.7–6.0 |
| lymphocytes (G/l) | 2.9±1.2 | 3.0±1.3 | 2.0–9.0 |
| monocytes (G/L) | 0.1±0.1 | 0.2±0.1 | 0–0.75 |
| **clinical chemistry** |  |  |  |
| total protein (g/L) | 60.9±4.0 | 62.1±3.8 | 60–79 |
| **clinical parameters** |  |  |  |
| pulse frequency (per min) | 101±19 | 114±30 | 70–140 |
| breathing frequency (per min) | 44±15 | 45±13 | 30-100 |
| temperature (°C) | 39.1±0.5 | 39.0±0.5 | 38.5–40.0˚C |

Legend: G- Giga; MCV – mean corpuscular volume; MCH – mean corpuscular hematocrit; MCHC – mean corpuscular hemoglobin concentration
